# TNFAIP8 Regulates Intestinal Epithelial Cell Differentiation and May Alter Terminal Differentiation of Secretory Progenitors

**DOI:** 10.3390/cells10040871

**Published:** 2021-04-12

**Authors:** Ryan Hood, Youhai H. Chen, Jason R. Goldsmith

**Affiliations:** Department of Pathology and Laboratory Medicine, Perelman School of Medicine, 422 Curie Blvd, Philadelphia, PA 19104, USA; ryanhood@sas.upenn.edu (R.H.); yhc@pennmedicine.upenn.edu (Y.H.C.)

**Keywords:** cell fate decisions, secretory progenitors, pseudotime, homeostasis, transcriptional regulation

## Abstract

The intestine is a highly proliferative dynamic environment that relies on constant self-renewal of the intestinal epithelium to maintain homeostasis. Tumor necrosis factor-alpha-induced protein 8 (TNFAIP8 or TIPE0) is a regulator of PI3K-mediated signaling. By binding to PIP2 and PIP3, TIPE family members locally activate PI3K activity while globally inhibiting PI3K activity through sequestration of membranous PIP2. Single-cell RNA sequencing survey of *Tipe0*^−/−^ small intestine was used to investigate the role of TIPE0 in intestinal differentiation. *Tipe0*^−/−^ intestinal cells were shown to shift towards an undifferentiated state, with the notable exception of goblet cells. Additionally, three possible novel regulators of terminal cell fate decisions in the secretory lineage were identified: Nupr1, Kdm4a, and Gatad1. We propose that these novel regulators drive changes involved in goblet cell (Nupr1) or tuft cell (Kdm4a and Gatad1) fate commitment and that TIPE0 may play a role in orchestrating terminal differentiation.

## 1. Introduction

Intestinal homeostasis relies on rapid and continuous self-renewal of the intestinal epithelium controlled by various inputs and signaling pathways. Intestinal stemness is regulated by the Wnt/β-catenin pathway, which maintains intestinal stem cell (ISC) self-renewal by mediating expression of ISC-specific genes [1]. The Wnt/β-catenin pathway is also involved in determining the secretory lineage in the absence of Notch signaling [2]. Additionally, mouse atonal homologue 1 (Math1 or Atoh1) is required for entry into the secretory lineage [3,4,5]. Activated Notch signaling, on the other hand, determines commitment to the absorptive lineage by upregulation of Hes1, an inhibitor of Atoh1. Terminal differentiation in the secretory lineage involves a number of different factors, including neurogenin 3 (Neurog3) in enteroendocrine cells [6,7,8], Kruppel-like factor 4 (Klf4) and sterile alpha motif pointed domain containing ETS transcription factor (Spdef) in goblet cells [9,10,11], growth factor independent 1 (Gfi1) in Paneth cells [12], and Gfi1b in tuft cells [13]. The balance between proliferation and differentiation is further mediated by a number of additional pathways, including Hedgehog, bone morphogenic protein (BMP), PI3K/Akt, Jak/STAT, and Eph-ephrin signaling pathways [14].

Tumor necrosis factor-alpha-induced protein 8 (TNFAIP8 or TIPE0) is a member of the TNFAIP8-like (TIPE) family of proteins along with TNFAIP8L1-3 (TIPE1-3) [15,16,17]. TIPE0, as well as other members of the TIPE family, has been found to interact with a variety of phospholipids, including phosphatidylinositol-4-phosphate (PI4P), PI (4,5) P2, PI (3,5) P2, PI (3,4) P2, and PI (3,4,5) P3 through a large hydrophobic TIPE homology (TH) domain [18,19,20]. Through binding to PIPs, TIPE0 plays an important role in modulating PI3K signaling. ADP-Glo PI3K assay demonstrated dose-dependent ATP consumption and potentiation of PI (3,4,5) P3 with addition of increasing concentrations of TIPE0, which suggests TIPE0′s ability to locally activate PI3K signaling [21]. Recently, however, mass ELISA in ileal and colonic samples and co-localization analysis using a PI (3,4,5) P3 biosensor demonstrated increased PI (3,4,5) P3 in the *Tipe0*^−/−^ intestine and in the cell membrane of *Tipe0*^−/−^ cells [22]. Global increase in PI (3–5) P3 also elevated PI3K/Akt signaling in *Tipe0*^−/−^ intestinal epithelium, suggesting that TIPE0 may globally suppress PI3K signaling [22]. Furthermore, aberrant PI3K/Akt signaling in the *Tipe0*^−/−^ intestine was found to alter the cellular composition of the intestinal epithelium, causing a shift towards an undifferentiated state [22]. In this study, we use this phenomenon of altered differentiation in the *Tipe0*^−/−^ gut, coupled to single-cell RNA-Seq pseudotime analyses, to discover novel regulators of secretory lineage fate commitment. 

## 2. Materials and Methods

### 2.1. Animals 

Wild-type (WT) C57BL/6 mice were purchased from the Jackson Laboratory (Bar Harbor, ME, USA) or bred in house. *Tipe0*^−/−^ mice were previously generated, as described elsewhere [23]. WT and *Tipe0*^−/−^ mice used in the study were colony controlled (from the same initial litter) but were propagated separately. All mice used in this study were housed under pathogen-free conditions in the University of Pennsylvania Animal Care Facilities. All animal protocols used were pre-approved by the Institutional Animal Care and Use Committee of the University of Pennsylvania. 

### 2.2. Single Cell RNA Sequencing Analysis

scRNA-seq dataset was generated by Goldsmith et al. [22]. All functions used in this study are either part of R base-functions or of the packages Monocle 3 [24,25,26], Dplyr, Matrix, or Tidyverse.

#### 2.2.1. Dimensionality Reduction Using PCA and UMAP

Pre-processing of droplet (10X) scRNA-seq data including demultiplexing, alignment to the mm10 transcriptome, and UMI-collapsing was performed by Goldsmith et al. [22]. 10X genomics output was loaded into R as a cell data set (CDS) object using the “load_mm_data” function from the Monocle3 package. A metadata column containing the short names of each gene was specified in addition to the 10X genomics output. The processed data was normalized by logarithmic and size factors to address differences in sequencing depth followed by dimensionality reduction by Principal Component Analysis (PCA) using the Monocle3 function “preprocess_cds”. Fourteen principal components were used as described in Goldsmith et al. [22]. The dimensionality of the data was further reduced for visualization by using Uniform Manifold Approximation and Projection (UMAP). This was implemented using the default parameters of the “reduce_dimension” function from the Monocle3 package. 

#### 2.2.2. Clustering and Annotation

Cells were grouped into clusters using Leiden-based community detection as reported by Levine et al. using the Monocle function “cluster_cells” [27]. This was performed with a k-parameter of 40 and a resolution of 1 × 10^−2^ (WT) or 4 × 10^−2^ (TKO, i.e., *Tipe0*^−/−^). Marker genes by cluster were determined using the Monocle function “top_markers” with the default number of cores and reference cells. Gene markers from Haber et al. were used to identify the cell types of each cluster [27]. Supplementary data from Haber et al. was reformatted to fit into a comma-delimited format and loaded into R. Then, a Monocle-3-specific function, “clust_marker_topx”, was written to generate a data frame that classifies top markers for each cluster by cell type according to Haber et al. First, the output of “top_markers” was filtered to exclude markers for which the fraction expressing was less than 0.10. Next, the gene name for all identified markers for a given cluster was stored as a vector. This vector was then iteratively compared to the cell type markers from Haber et al., and similar markers were stored in a data frame whose columns corresponded to the associated cell type. Using this function, the cell type identities of each cluster were determined. Cells were then annotated by storing a character vector as a column of “colData” of the CDS object. Initial annotation was sufficient for the WT CDS. However, the TKO UMAP plot lacked clusters with markers of IELs and enteroendocrine cells. These cell types were identified by plotting IEL or enteroendocrine marker gene expression on the UMAP plot. These cells were then visually subsetted using “choose_cells” and annotated as described above. Furthermore, substructure in clusters 3 and 10 was identified by visually subsetting the individual clusters as described above and clustering these cells separately. Further annotations were then added to the CDS as above.

#### 2.2.3. Determining Cell Type Percentages

A Monocle-3-specific-function, “cell_type_percentages”, was written to determine the number of cells in each subset by assigned cell type. First, the length of the vector “rownames” of “colData”, which contains the cell barcodes, was determined representing the total number of cells in the CDS. Next, this was done for the “colData” matrix subsetted by cell type representing the total number of cells of each type. The number of cells of each subtype was then divided by the total number of cells and multiplied by 100, yielding the percentage of the total composed of each cell type. 

#### 2.2.4. Learning Trajectories and Ordering Cells in Pseudotime

Trajectories were learned by Monocle 3′s reversed graph embedding algorithm using the function “learn_graph” [26]. Subsequently, cells were ordered in pseudotime using the function “order_cells” with a selected node representing Lgr5+ stem cells to define the 0 point of pseudotime.

#### 2.2.5. Pseudotime Binning Analysis

A Monocle-3-specific function was written to subset cells by pseudotime, fit a regression to each by genotype, and generate a data frame with the significant differences in expression for identified genes by pseudotime bin. This was done by storing each cell’s pseudotime value to the “colData” matrix. The CDSs of each genotype were combined using the function “combine_cds”, which automatically adds sample (in this case genotype) to the “colData” of the combined CDS. The combined CDS was input to the function “pseudotime_bin_analysis”. First, the combined CDS was subsetted by single-unit pseudotime bins and a regression model was fit to each gene to compare expression between genotypes. The output was then filtered to remove intercept values and non-significant genes. Genes differentially expressed across different pseudotime bins were stored as a vector, excluding repeating values. A data frame was then generated, with the first column containing the short names of identified genes and the following columns containing NA values. These NA values were then replaced with the significant regression values for each gene. The function then returned a list of the generated data frame as well as the individual outputs for each pseudotime bin.

#### 2.2.6. Secretory Branch Analysis

The secretory branch was identified by plotting the secretory precursor Atoh1 on the UMAP plot for each genotype. The branch was isolated using the “choose_cells” function from Monocle 3 and manually selecting the cells along the trajectories identified above. Regression analysis was performed on each genotype separately using genotype as the independent variable. The regression for each genotype was then compared by student’s *t*-test. Gene expression was plotted across pseudotime using the function “plot_genes_in_pseudotime”.

## 3. Results

### 3.1. Loss of TIPE0 Alters Intestinal Differentiation

**Figure 1:**
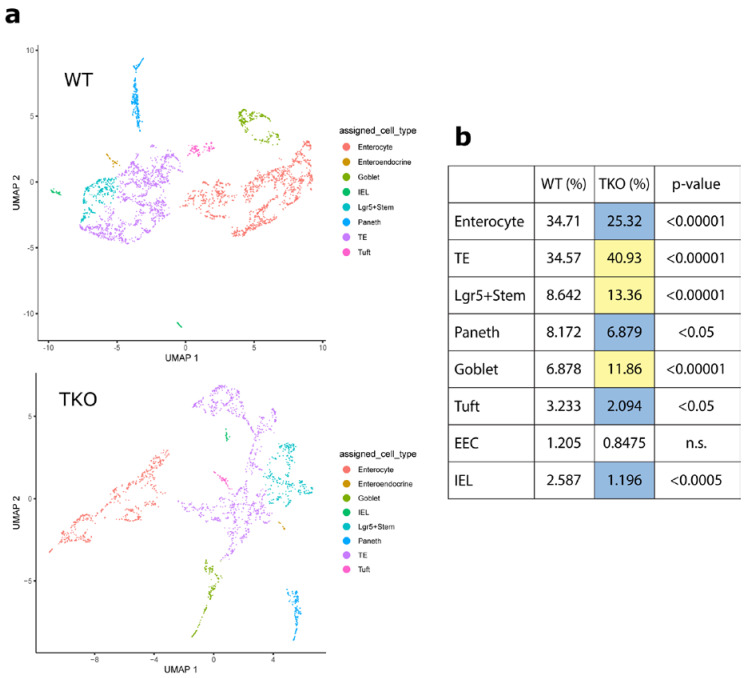
TKO (*Tipe0*^−/−^) intestine is shifted towards an undifferentiated state. (**a**) Uniform Manifold Approximation and Projection (UMAP) plots colored by cell type, *n* = 3 mice/genotype, wild-type (WT): 3402 cells, TKO: 2006 cells; (**b**) Tabulated percentages of cell types, colored by difference between TKO and WT (purple (negative), yellow (positive)). Significance determined by *z*-test for independent proportions. n.s. = not significant.

Single-cell RNA sequencing (scRNA-seq) was used to confirm the effect of TIPE0 knockout on the composition of the ileal epithelium. Monocle 3 was used to cluster cells at a resolution of 1 × 10^−2^ for WT (*n* = 3402) and 4 × 10^−2^ for *Tipe0*^−/−^ (*n* = 2006). Larger resolution values result in more clusters [28], which was necessary for proper determination of cell types in the smaller dataset. Seventeen clusters were identified for each genotype, and two additional clusters were identified in the *Tipe0*^−/−^ dataset (representing intraepithelial lymphocytes (IELs) and enteroendocrine cells (EECs)). Additionally, substructure was identified for clusters 3 and 10 representing tuft cells and another cluster of goblet cells, respectively. Clusters were then annotated using established gene profiles for the small intestinal epithelium. Clusters representing each of the cell types found in the ileal epithelium were identified as well as a cluster in each sample representing intraepithelial lymphocytes (Figure 1a). *Tipe0*^−/−^ mice had similar or decreased levels of terminally differentiated enterocytes and increased levels of transitional/partially differentiated and Lgr5+ stem cells, consistent with previous studies (Figure 1b) [22]. Goblet cells were significantly enriched in the *Tipe0*^−/−^ intestine, which is also consistent with previous findings by Goldsmith et al. These data further suggest that differentiation in the *Tipe0*^−/−^ intestine is dysregulated, with a shift from terminally differentiated enterocytes towards Lgr5+ stem cells and transitional enterocytes with the notable exception of goblet cells.

### 3.2. Loss of TIPE Leads to Altered Gene Expression in the Intestinal Epithelium

**Figure 2:**
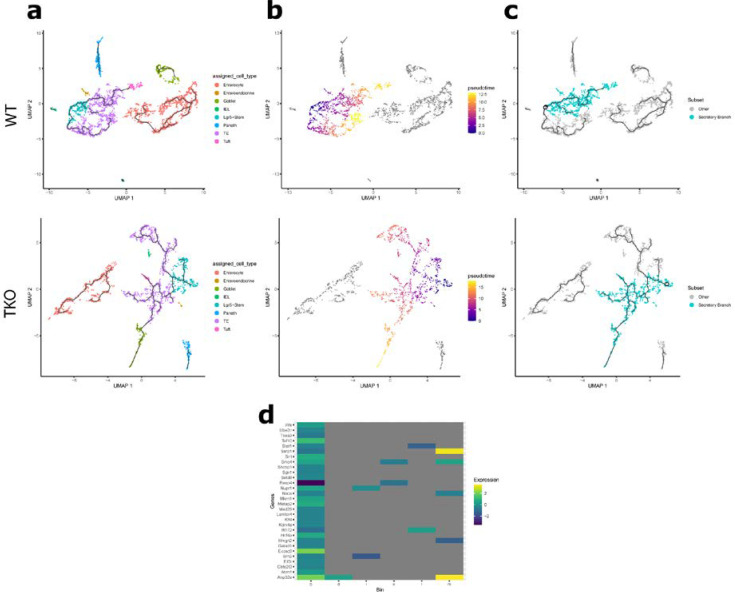
Identifying differentially expressed genes through pseudotime binning. (**a**–**c**) UMAP plots, black lines represent branched cell trajectories; (**a**) cells colored by cell type; (**b**) cells colored by pseudotime; (**c**) cells colored by selected branch for downstream analysis (blue); (**d**) heatmap of regression analysis slope values (β_0_) by genes determined to regulate expression by gene ontology analysis. *p* < 0.05 for all genes shown.

Given that TIPE0 modulates PI3K/Akt signaling and that expression of stem cell markers and beta-catenin targets are altered by loss of TIPE0, we investigated which genes may be responsible for the changes in the composition of the *Tipe0*^−/−^ intestine. Monocle 3 was employed to construct single-cell trajectories that reflect the kinetics of various gene modules (Figure 2a). Based on these trajectories, cells were then ordered in pseudotime, defining the node at Lgr5+ stem cells (Figure 2b). Due to technical limitations, regression analysis using both genotype and pseudotime as continuous independent variables could not be performed. Thus, pseudotime was grouped into one-unit bins and regression using genotype was performed for each bin. This analysis revealed 298 differentially expressed genes between WT and *Tipe0*^−/−^ through different points in pseudotime. Thirty genes were then selected for further analysis based on capacity to regulate transcription or modulate signaling pathways involved in intestinal epithelial cell differentiation (Figure 2d). However, for the identified genes, differential expression was restricted to early time points with no significant difference at later time points. This was likely due to the varying trajectories that composed each point in pseudotime following differentiation of Lgr5+ stem cells into secretory and absorptive precursors that would affect the measure level of expression at a given time point. Thus, using the genes identified by this analysis, it was necessary to select a single major branch for further analysis (Figure 2c). Despite this, these data further suggest that TIPE0 is involved in modulating expression programs that regulate differentiation in the intestine.

### 3.3. Nupr1, Kdm4a, and Gatad1 Are Possible Novel Regulators of Secretory Cell Fate

**Figure 3:**
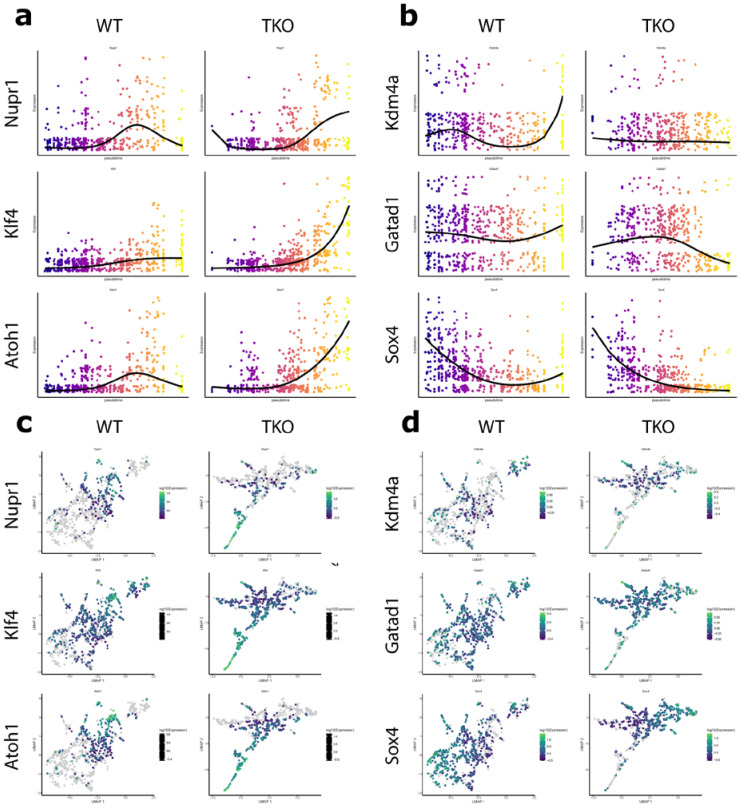
Genes differentially expressed across psuedotime in the secretory branch of TKO. (**a**,**c**) Plots of expression vs. pseudotime for genes upregulated (**a**) or downregulated (**c**) in TKO. Line represents the polynomial regression of expression vs. psuedotime. Cells are shown, colored by pseudotime (blue to yellow). Difference between the slope of TKO and WT fits is shown along with p-values; (**b**,**d**) UMAP of cell data set branch subsets. Cells are colored by expression of genes upregulated (**b**) or downregulated (**d**) in TKO. Cells are colored by gene expression.

To investigate the kinetics of transcriptional regulators across pseudotime, the branch representing secretory precursors was chosen for further analysis based on the enriched goblet cell count in *Tipe0*^−/−^ intestine. Following subsetting, expression of each gene was fit to pseudotime across the branch in either genotype and the fits between each genotype were compared. This identified nine genes that were differentially expressed both across pseudotime within each genotype and between genotypes. Five genes (Nupr1, Klf4, Atoh1, Kdm4a, and Gatad1) were identified in having roles specific to differentiation (Figure 3). Plotting the polynomial fit of expression across pseudotime revealed that most of these genes demonstrated a point at which the slope of *Tipe0*^−/−^ diverged dramatically from that of the WT (Figure 3a,c). This change could represent a switch between expression programs responsible for cell fate decisions. Notably, in the TIPE^−/−^ UMAP plot, goblet cells are at the end of the secretory trajectory whereas in that of the WT, tuft cells are the end of the trajectory. Therefore, it is possible that the differential expression of these genes is due to this difference in clustering. However, this does not rule out the possible role of TIPE0. Expression of proteins known to regulate cell fate decisions demonstrate a similar fit, supporting the possible role of the identified genes in this process. For example, Spdef causes expansion of goblet cells and a reduction of Paneth cells when overexpressed in the small intestine [11]. The plot of Spdef expression vs. pseudotime demonstrates a remarkably similar shape to the plots in Figure 3a,c (data not shown). Additionally, conditional loss of Sox4 resulted in loss of tuft and enteroendocrine cells and impaired tuft cell hyperplasia after helminths infection [29]. The plot of Sox4 expression vs. pseudotime also demonstrates a similar shape to the plots of Gatad1 and Kdm4a, despite its high expression in ISCs. 

Lastly, we attempted to validate the findings using RT-qPCR of previously generated small intestinal WT and *Tipe0*^−/−^ RNA samples for changes in Nupr1, Kdm4a, and Gata1 between them; however, the results were inconclusive (data not shown). We believe that the cell-specific expression of these genes causes differences in their expression to be drowned out in the analysis of pooled cells. 

## 4. Discussion

Loss of TIPE0 alters intestinal patterns of differentiation, generally shifting cells towards a more undifferentiated state. The intestinal epithelium in *Tipe0*^−/−^ is enriched in Lgr5+ stem cells and partially differentiated cells, whereas terminally differentiated cells such as Paneth, tuft, and enteroendocrine cells are reduced. Goblet cells are the exception to this and are more abundant in the *Tipe0*^−/−^ intestinal epithelium. Interestingly, the clustering of WT and *Tipe0*^−/−^ cells yielded secretory trajectories that terminate in different cell types. This allowed for the investigation of regulators of cell fate decisions that produce each of these cell types as well as the role TIPE0 may play in this process.

Both Atoh1 and Klf4 have been shown to be involved in goblet cell differentiation, supporting the data found in this study. Loss of Atoh1 (also named Math1) in mice led to depletion of secretory cells (goblet, Paneth, and enteroendocrine) without affecting enterocytes. Additionally, co-localization of Atoh1 and Ki-67 in some proliferating cells suggests that secretory cells arise from a shared lineage of Atoh1-expressing precursors [3]. Intestine-specific knockout of Atoh1 yielded a similar loss of secretory cells [4]. *Klf4*^−/−^ mice demonstrated a 90% decrease in goblet cell count while all other epithelial cell types remained constant [9]. Intestine-specific deletion of Klf4 also led to failure of goblet cell differentiation [30]. Recently, specific deletion of Klf4 in Bmi+ reserve stem cells along with parallel lineage tracing led to a reduction in goblet cells in the Bmi+ lineage, suggesting that Klf4 is able to regulate goblet cell differentiation in Bmi+ stem cell lineages [10]. 

Nupr1, however, is less clearly associated with goblet cell development. Nupr1 is a transcription factor responsible for mediating stress-responses. It has been found to play a role in pancreatic tumorigenesis as well as in the protection of cancer cells from stress induced death by inhibiting apoptosis [31]. Additionally, loss of Nupr1 led to decreased expression of endoplasmic reticulum stress response associated genes [32]. In the small intestine, researchers found that Nupr1 might play a role in Paneth cell differentiation and function using single-cell genomics [33]. However, the data from this study suggest that Nupr1 expression precedes differentiation to Paneth cells and is also present in Goblet cells. This may indicate that Nupr1 plays a role in directing goblet/Paneth precursor differentiation. Interestingly, Nupr1 has been found to bind to RING1B, part of the Polycomb repressive complex 1 that has been shown to promote intestinal stem cell self-renewal [34,35]. Despite this, the exact role Nupr1 plays in cell fate decisions remains unclear. 

The distinct requirements for tuft cell differentiation have yet to be determined. Intestine-specific knockout of Atoh1 has been shown to either increase [13] or ablate [36] tuft cells in the small intestine. Inhibition of Notch signaling, which increases Atoh1 activity, was also shown to increase the number of tuft cells in the intestine [37]. This study suggests a model in which tuft cells arise from secretory precursors, in agreement with the findings of Gerbe, F. et al. [36] and VanDussen, K. et al. [37], and subsequent loss of Atoh1 expression is involved in determination of tuft cells. This is supported by the trajectory in the WT in which the immediate precursors to tuft cells are Atoh1+. Additionally, Sox4 has been identified as a possible regulator of tuft and enteroendocrine cell differentiation [27]. This study identifies two other proteins that may play a role in Tuft cell differentiation: Kdm4a and Gatad1. Kdm4a has been identified as a H3K9Me3 and H3K36Me3 specific demethylase [38,39]. It has already been shown to regulate cell fate decisions in skeletal muscle cell, pancreatic cell, and embryonic stem cell differentiation [40,41,42]. Gatad1 has been proposed to function as a histone deacetylase [43]. However, recent work has shown that it may be part of a transcriptionally repressive histone complex, regulating transcription through indirect interaction with H3K4Me3 through Kdm5a [44]. Gatad1 has also been shown to regulate mammalian lens and liver cell differentiation [45,46]. Additionally, Gatad1 was found to modulate PI3K/Akt signaling activity in hepatocellular carcinoma cells through upregulation of phosphatase of regenerating liver 3 (PRL3) by binding its promoter [47]. Both Kdm4a and Gatad1 represent possible novel regulators of tuft cell differentiation through epigenetic modulation of secretory precursors. The precise mechanism of their regulation and role in this process has yet to be determined. Additionally, it must be noted that increased baseline inflammation in the *Tipe0*^−/−^ intestine may influence changes in the stem cell niche and differences seen in terminal differentiation [22]. Further, without heterozygous littermate controls, differences in the composition of the intestinal microbiome may have influenced patterns of differentiation as well. Although the mechanism of the differential expression of Nupr1, Gatad1, and Kdm4a in the WT and *Tipe0*^−/−^ requires further investigation, each proposed regulator’s trajectory is consistent with a role in the terminal differentiation of either goblet or tuft cells. Further work will also need to be done to validate these findings, first by sorting populations of goblet and tuft cells from WT and *Tipe0*^−/−^ ileums and then by performing downstream RT-PCR and protein analyses.

The role of TIPE0 in modulating PI3K/Akt signaling, as well as the observed changes in the cellular composition of the *Tipe0*^−/−^ intestinal epithelium, suggest that TIPE0 may play a role in the modulation of intestinal epithelial cell differentiation. The shift towards undifferentiated cells in *Tipe0*^−/−^ likely results both from increased proliferative signaling by the PI3K/Akt pathway as well as crosstalk with the Wnt/B-catenin pathway that promotes stemness. However, how this pathway modulates later cell fate decisions is currently unknown. 

This study has further supported the effect of TIPE0 knockout on intestinal epithelial composition and in mediating changes in expression patterns that alter intestinal epithelial cell differentiation. This study also has identified three possible novel regulators of terminal secretory differentiation: Nupr1 in goblet cell differentiation and both Kdm4a and Gatad1 in tuft cell differentiation. 

## Data Availability

The data presented in this study are available from Goldsmith, J.; et al. at doi:10.1038/s41467-020-16379-2, or on request from the corresponding author.
